# Acute Effects of Kinesiology Taping Stretch Tensions on Soleus and Gastrocnemius H-Reflex Modulations

**DOI:** 10.3390/ijerph18094411

**Published:** 2021-04-21

**Authors:** Yung-Sheng Chen, Shi Zhou, Zachary J. Crowley-McHattan, Pedro Bezerra, Wei-Chin Tseng, Che-Hsiu Chen, Xin Ye

**Affiliations:** 1 Department of Exercise and Health Sciences, University of Taipei, Taipei 111, Taiwan; yschen@utaipei.edu.tw (Y.-S.C.); speedceng@gmail.com (W.-C.T.); 2 Exercise and Health Promotion Association, New Taipei City 241, Taiwan; 3 Faculty of Health, Southern Cross University, Lismore 2480, Australia; shi.zhou@scu.edu.au (S.Z.); zac.crowley@scu.edu.au (Z.J.C.-M.); 4 Escola Superior Desporto e Lazer, Instituto Politécnico de Viana do Castelo, Rua Escola Industrial e Comercial de Nun’Álvares, 4900-347 Viana do Castelo, Portugal; pbezerra@esdl.ipvc.pt; 5 The Research Centre in Sports Sciences, Health Sciences and Human Development, 5001-801 Vila Real, Portugal; 6 Department of Sport Performance, National Taiwan University of Sports, Taichung 404, Taiwan; jakic1114@ntupes.edu.tw; 7 Department of Rehabilitation Sciences, University of Hartford, West Hartford, CT 06117, USA

**Keywords:** kinesiology taping, motoneuron excitability, motor control, calf muscle, Hoffmann-reflex

## Abstract

This study examined the acute effects of stretch tensions of kinesiology taping (KT) on the soleus (SOL), medial (MG), and lateral (LG) gastrocnemius Hoffmann-reflex (H-reflex) modulation in physically active healthy adults. A cross-over within-subject design was used in this study. Twelve physically active collegiate students voluntarily participated in the study (age = 21.3 ± 1.2 years; height = 175.6 ± 7.1 cm; body weight = 69.9 ± 7.1 kg). A standard Y-shape of KT technique was applied to the calf muscles. The KT was controlled in three tension intensities in a randomised order: paper-off, 50%, and 100% of maximal stretch tension of the tape. The peak-to-peak amplitude of maximal M-wave (M_max_) and H-reflex (H_max_) responses in the SOL, MG, and LG muscles were assessed before taping (pre-taping), taping, and after taping (post-taping) phases in the lying prone position. The results demonstrated significantly larger LG H_max_ responses in the pre-taping condition than those in the post-taping condition during paper-off KT (*p* = 0.002). Moreover, the ΔH_max_/M_max_ of pre- and post-taping in the SOL muscle was significantly larger during 50%KT tension than that of paper-off (*p* = 0.046). In conclusion, the stretch tension of KT contributes minor influence on the spinal motoneuron excitability in the triceps surae during rest.

## 1. Introduction

The gastrocnemius and soleus (SOL) muscles are the main skeletal muscles in controlling plantarflexion (PF). The physiologic features and functional roles are specifically different among the calf muscles. It has been reported that the architectural features (line of action, angle of pennation, fascicle lengths) [1], muscle spindle activation [2], and mechanical output [3] are different between the gastrocnemius and SOL muscles. The functional role of the gastrocnemius is to maintain mechanical output during locomotion and exercises, whereas the SOL predominately plays a role to stabilise the ankle joint position during postural control [3]. 

Kinesiology tape (KT) has been extensively used by sports practitioners and physical therapists. This tape is designed with the elastic nature of polymer materials and specific tape structure to improve skin tissues and neuromuscular functions. However, the effectiveness of KT application to physiological performance and clinical benefits is controversial in the literature [4,5,6]. For example, Tremblay and Karam [7] reported that KT application has limited effects on increased corticospinal excitability at rest, as evidenced by the motor-evoked potentials. Another study also reported no effect of KT on the vastus medialis H-reflex during maximal voluntary knee extension when 25% KT stretch tension was applied [8]. Magalhães et al. [9] also reported no therapeutic effect of KT application on the soleus (SOL) H-reflex after maximal voluntary contractions when facilitation and inhibitory directions with 30% stretch tension were applied to physically active adults. In contrast, optimal benefits of KT application were observed during a sensorimotor synchronisation test when light (15–25% of the maximal tension) and moderate (40% of the maximal tension) KT stretch tensions were applied to the wrist muscles, compared to non-tension KT [10,11]. Moreover, an increase in magnitude of SOL H-reflex modulation was observed when 15–25% of KT maximal stretch tension on musculotendinous junction was applied [12]. At this time, research findings are controversial on the use of KT.

In his KT manual, Dr. Kenzo Kase, the inventor of KT tape, recommends a 50% maximal KT stretch tension to optimise functional performance [13]. The application of 30–40% maximal KT tape tension improves ankle movement stability [14], whereas KT with 15–20% of maximal KT stretch tension is not beneficial on proprioception during reproduction of the joint position sense test [15]. We recently found that test–retest reliability of joint position sense test of the dorsiflexors depends upon the stretch tension of KT [16]. Moderate intensity of KT taping produces optimal reproducibility of joint position sense measurement in the dorsiflexors. Thus, it seems that the application of KT on peripheral sensory activation may depend upon the intensity of the tape tension. However, we recently reported that there was no change of SOL H-reflex modulation during standing and lying prone positions when different KT tape tensions were applied [17]. To the best of our knowledge, no study has examined the effect of KT application on H-reflex modulation in synergist muscles.

The purpose of this study was to investigate the effects of KT stretch tensions on the SOL, medial gastrocnemius (MG), and lateral gastrocnemius (LG) H-reflex modulation. The intensity of KT stretch tensions included no tension (paper-off), 50% of maximal stretch tension (50%KT_max_), and 100% of maximal stretch tension (100%KT_max_). We hypothesised that the maximal stretch of KT could result in increases in the excitability of SOL, MG, and LG motoneuron pool and could enlarge the size of SOL, MG, and LG H-reflex modulation.

## 2. Materials and Methods

### 2.1. Participants

Based on a previous study conducted by Firth et al. [12], we conducted an a priori power analysis using the G*Power software (G*Power version 3.1.9.4, Düsseldorf, Germany) [18], on the basis of the possible large effect size (f = 0.40) for the difference in three tape tensions, an α level of 0.05, and a power (1-β) of 0.80. It was shown that at least 12 participants were necessary (F-test ANOVA, repeated measures, within factors).

Twelve healthy and physically active men were recruited to participate in this study (Table 1). The inclusion criteria of participations included (1) regular exercise (either aerobic or resistance exercise) at least 3 times a week with an accumulation of 150 min/week; (2) between 20 and 30 years old. Exclusion criteria included any history of severe neuromuscular injury, current lower extremity injury, and neurological diseases. The international physical activity questionnaire (IPAQ) [19] was used to estimate the weekly physical activity of the participants. All participants signed an informed consent form and undertook a familiarisation session prior to the experiment. Participants were required to refrain from any strenuous activity 24 hours before participation. This study was approved by the human ethics committee of the University of Taipei (UT-IRB-2017-049) and was conducted in according to the Declaration of Helsinki. This study has been registered in ClinicalTrials.gov with an identifier of NCT03865251.

### 2.2. Experimental Procedure

All assessments were conducted in an exercise performance laboratory at the University of Taipei. The participants first visited the laboratory for a familiarisation protocol. During the first visit, participants completed initial anthropometric data and practiced the testing protocol. The second, third, and fourth visits were trials of paper-off, 50%KT_max_, and 100%KT_max_ conditions. The experimental conditions were randomised in the sequence order via Research Randomizer (https://www.randomizer.org/, accessed on 12 April 2021) [20] with at least a 2-day interval between consecutive visits.

**Figure 1:**
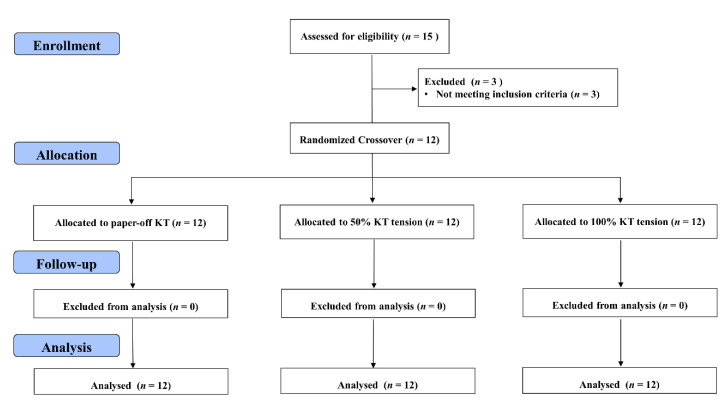
Experimental flow diagram of the study.

For each experimental condition, the H/M recruitment curve was carried out in lying prone positions in pre-taping, taping, and post-taping periods. After the pre-taping condition assessment, KT was applied to the participants’ dominant leg. The taping application was conducted by a qualified physical therapist (P.C.S) throughout the experiment. The post-taping H-reflex assessment was conducted about 1 minute after removing the KT taping. The duration of the experiment for each visit was around 90–120 min (Figure 1).

### 2.3. Kinesiology Taping

The experimental conditions were set to paper-off, 50%KT_max_, and 100%KT_max_. Y-shaped kinesiology tape with inhibitory direction was applied to the target locations, from the calcaneal tuberosity to the lateral condyle of the femur and the medial condyle of the femur. The first strip of the KT (Nitto Kinesiology Tape, NKH-X, Japan) was applied alongside the board of lateral side of the gastrocnemius and the peroneus longus muscles. Another strip of the KT was placed alongside the board of the MG and the SOL muscles. During the taping process, the participants lay down in a prone position, and they fully extended their knee joint and maintained the ankle joint in a full dorsiflexion position. The KT was facilitated by a qualified physical therapist and the KT tensions were manipulated according to individual calf length. The application of KT was processed in accordance with Kenzo Kase’s KT manual.

### 2.4. Surface Electromyography

Surface EMG electrodes (TSD150B, Biopac Systems, Goleta, CA, USA) were used to record the reflex responses in the SOL, MG, and LG muscles. The electrode housing contained two stainless steel silver bars spaced 20 mm apart and with built-in amplification. In preparation, the participants were instructed to wear shorts and no shoes. The dominate leg was used for assessment and was determined as the preferable leg to kick a ball. The participants first lay on a massage table in the prone position with their feet hanging off the end of the table. Additionally, their knee joint was maintained straight in a comfortable position. The applications of the electromyographic (EMG) electrode placements for MG, LG, and SOL and electrical electrodes were prepared by a researcher, following by cleaning with alcohol wipes and skin preparation. All locations of EMG placements were determined in accordance with the recommendations of SENIAM [21]. The electrode placement on the MG was the location with most prominent bulge of the muscle belly through the palpating examination. The electrode placement on the LG was set at one-third of the distance between the head of the fibula and the calcaneal tuberosity. The EMG sensor to record the SOL was placed at two-thirds of the distance from the medial condyles of the femur to the medial malleolus of the tibia and central to the medial–lateral direction of the SOL muscle border. A waterproof pen was used to ensure the same electrode locations in repeated measures. A reference electrode was placed on the medial malleolus of the non-dominant leg. Adhesive tapes were used to secure the electrodes on the skin. 

A data acquisition system (MP160, Biopac, CA, USA) was used to process the EMG signals, filtered with a band-pass range of 20–450 Hz and amplified with a gain of 1000 times. Sampling rate of analogue to digital conversion was set to 2.5 kHz.

### 2.5. H-Reflex Measurement

For the H-reflex measurement, a single electrical impulse was applied to the posterior tibial nerve to elicit the H-reflex and M-wave responses in the SOL, MG, and LG muscles via an electrical stimulator with 1000 µs pulse duration and maximal output voltage set at 400 V (DSH7, Digitimer, Welwyn Garden City, Herfordshire, UK). The optimal location of a cathode electrode to elicit the SOL H-reflex response was carefully identified by the principal investigator. A reusable rubber-based self-adhesive electrode (10 × 10 mm, FA 25, Gem-Stick, Australia) was placed on the popliteal fossa as the cathode and another square shape of reusable rubber-based self-adhesive electrode (50 × 50 mm, Life Care, New Taipei City, Taiwan) was fixed over the patella as the anode. A commercial data acquisition and analysis system (Acqknowledge 5.0, Biopac, CA, USA) was used to synchronise the electrical stimulations and the EMG records. To establish the recruitment curve, stimulation intensity was increased with 10 mA increments from the H-reflex threshold until the maximal peak-to-peak amplitude of the M-wave (M_max_) was identified. The stimulation intensity to elicit the M_max_ was the supra-maximal stimulation with 1.5 times of stimulation intensity while the first plateau intensity of the M_max_ was identified. The maximal peak-to-peak amplitude of the H-reflex response (H_max_) was then determined by using 2 mA increments from the stimulation intensity of the threshold of H response in the subsequent determination. Each stimulation intensity was applied four times with 8–10 s interval to avoid potential effects of post-activation depression [22]. The number of stimuli for the determination of the H/M recruitment curve varied from individual to individual, with a range of 40–60 stimuli in each experimental condition. To avoid H-reflex modulation compromised to the knee joint angle, participants were asked to fully extend their knee joint angle on a massage table in a comfortable and relaxed position [22].

### 2.6. Data Analyses

The peak-to-peak amplitudes of H_max_ and M_max_ were measured off-line for the respective experimental conditions. The average value of H-reflex and M-wave variables in all successful trials were processed by one researcher. The H_max_, M_max_, and H_max_/M_max_ ratio were calculated for statistical analyses. The normalisation of the H_max_/M_max_ ratio can be used to indicate the proportion of spinal motoneuron pool activated by the Ia afferent inputs during the reflex response [22]. The relative changes of the reflex responses over time segments were measured as ΔH_max_, ΔM_max_, and ΔH_max_/M_max_ ratio.

### 2.7. Statistical Analyses

Descriptive data of the measured variables were presented as mean values and standard deviations (mean ± SD). The normal distribution of study variables was examined with the Kolmogorov–Smirnov test. Two-way repeated measurement of analysis of variance test (ANOVA) [Tension (3) × Time (3)] was used to examine the dependent variables for each muscle. When a significant main effect or an interaction was found, a post hoc analysis with Bonferroni adjustment was conducted to identify significant differences between the values. Intra-class correlation coefficients (ICC_2,1_) with a two-way random model and absolute agreement were performed to determine reliability of the measure among the pre-KT condition in the second, third, and fourth visits. The level of ICC values was expressed as nearly perfect (0.9–1), very large (0.70–89), large (0.50–69), moderate (0.31–49), or small (0–0.3) [23]. An alpha value of *p* ≤ 0.05 was set for significant differences between the means. All statistical analyses were performed using SPSS version 25.0 software for windows (IBM, Armonk, NY, USA).

## 3. Results

### 3.1. Physical Characteristics of the Participants

The physical profiles of the participants are shown in Table 1. The participants recruited in the present study were physically active adults, as demonstrated by high values of the IPAQ score.

### 3.2. Reliability Measurement

Reliability of the H_max_, M_max_, and H_max_/M_max_ in three pre-taping condition of measurements is shown in Table 2. The results showed a high level of reliability of the H_max_ (ICC = 0.93, nearly perfect, to 0.96, nearly perfect), M_max_ (ICC = 0.88, very large, to 0.92, nearly perfect), and H_max_/M_max_ (ICC = 0.81, very large, to 0.91, nearly perfect) in the SOL, MG, and LG muscles.

### 3.3. M-Wave and H-Reflex Responses

**Figure 2:**
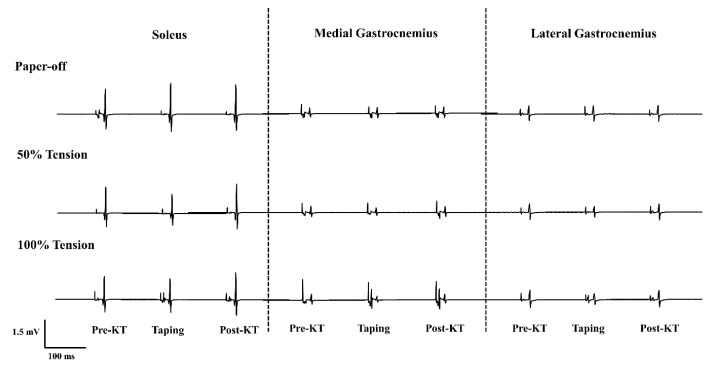
Raw electromyograph of the soleus, medial and lateral gastrocnemius H-reflex from one representative participant in paper-off, 50%KT_max_, and 100%KT_max_ conditions.

For the SOL muscle, a significant interaction between stretch tension and time was observed in the M_max_ (*F*_2__,__22_ = 3.70; *p* = 0.011). Post hoc analysis revealed that larger M_max_ was only found in 50%KT_max_ than that in paper-off in the pre-taping condition (paper-off = 2.80 ± 0.45 mV vs. 50%KT_max_ = 3.05 ± 0.49 mV; *p* = 0.038). Meanwhile, the MG demonstrated no statistical differences of the H_max_, M_max_, and H_max_/M_max_ in all comparisons. For the LG muscle, a significant interaction was identified (*F*_2__,__22_ = 2.97; *p* = 0.030) for the H_max_. Post hoc analysis revealed that a larger H_max_ was found in the pre-taping condition than that of the post-taping condition in the paper-off stretch tension (pre-taping = 0.75 ± 0.39 mV vs. post-taping = 0.69 ± 0.37 mV; *p* = 0.002) (Table 3). Figure 2 shows a raw EMG of H-reflex responses recorded in the SOL, MG, and LG in all testing conditions from one representative.

### 3.4. ΔM-Wave and ΔH-Reflex Responses

**Figure 3:**
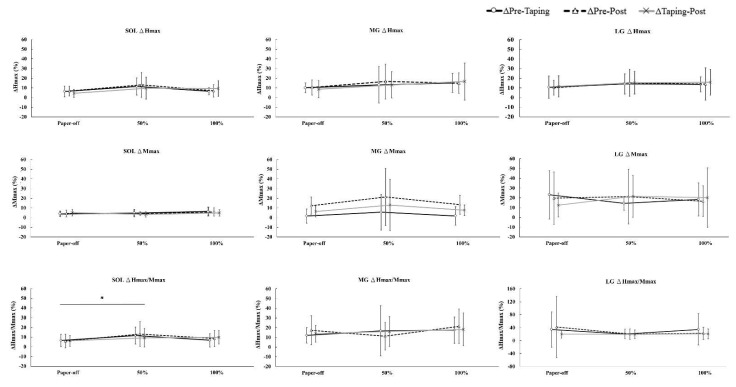
Percentage changes in pre-taping, pre-post, taping-post in paper-off, 50%KT_max_, and 100%KT_max_. * Significant difference in pre-post-between paper-off and 50% stretch tension (*p* < 0.05).

In the SOL muscle, a significant main effect for stretch tension was observed in the ΔH_max_/M_max_ parameter (*F*_2__,__22_ = 4.94; *p* = 0.035; η^2^ = 0.31). The post hoc analysis revealed statistical differences of pre-and-post difference between paper-off and 50%KT_max_ (paper-off = 6.68 ± 7.17% vs. 50%KT_max_ = 13.99 ± 13.11%; *p* = 0.046). In contrast, no significant difference of interaction and main effects were found in the MG and LG muscles (Figure 3).

## 4. Discussion

This was the first study to report the acute effect of KT stretch tensions on spinal motoneuron excitability in the SOL, MG, and LG muscles. The primary finding of the study was that pre- and post-comparison was only found significantly in the LG H-reflex modulations when the paper-off KT application was carried out. The secondary finding of the study was that the SOL H-reflex response demonstrated the stretch tension effect on ΔH_max_/M_max_ when paper-off and 50%KT_max_ in pre- and post-taping differences were compared. However, the KT application did not contribute to facilitatory or inhibitory effects on the MG H-reflex modulation despite the intensity of KT stretch tension.

The present study revealed that pre-and-post taping conditions were significantly different in the LG H-reflex modulation when paper-off was applied. Interestingly, there was no time effect on H-reflex modulation in other comparisons. Martínez-Gramage et al. [24] reported that KT can reduce the duration of LG EMG activity during walking with KT applied to the gastrocnemius for 72 hours (KT application to 50–75% maximal stretch tension with inhibitory technique). The short duration of motoneuron activation of the LG muscle during gait may be related to the inhibitory effects of skin stretch receptors. Another study conducted by Davison et al. [25] during a single vertical jump movement also supported this notion. Moreover, Magalhães et al. [26] reported that KT with 30% of maximal stretch tension applied to the LG and MG muscles lasting 48 hours can improve the rate of force development, but not the peak force of muscle strength. It seems that KT application could potentially regulate neural mechanisms during motor tasks. The underlying mechanisms to alter the motor activities with KT application are possibly related to the changes in neural inputs of cutaneous receptors facilitated by KT application on the calf muscles [27]. Indeed, the spinal motoneuron activation can be mediated by the changes in cutaneous sensory feedback [28]. However, our study showed that manipulation of KT to the triceps surae provided no influence on proprioceptive feedback, as evidenced by the H-reflex assessment. Thus, the cutaneous mechanoreceptors may not be a primary factor to regulate H-reflex modulation in the present study. 

It is interesting to note that the significant difference of ΔH_max_/M_max_ was only found between paper-off and 50%KT_max_ in the SOL muscle. The main purpose of this study was to examine the acute effect of KT on H-reflex modulation in the triceps surae. Sensorimotor inputs from the muscle spindle are supposed to serve as the main neural mechanisms to modulate motoneuron excitability at the spinal level. Muscle spindle feedback differs between the SOL and gastrocnemius in humans due to diverging motor unit recruitment thresholds and the number of muscle spindles [29]. Tucker and Tücker [2] demonstrated that the amplitude of the SOL H-reflex response was larger than that of the H-reflex response in the gastrocnemius during rest and submaximal and maximal muscle contractions, indicating different motor unit recruitments from Ia afferent inputs between the muscles. The different characteristics of motor adaptation to KT stretch tensions between the SOL and gastrocnemius muscles may be related to the sensitivity of peripheral sensory inputs to the mechanical stimulation of KT [3].

No change of ΔH_max_/M_max_ ratio in the MG and LG muscles indicated no KT stretch tension effect on the gastrocnemius reflex responses. It was hypothesised that the proprioception and sensorimotor function would be increased when the KT is stretched to a greater tension to target muscle or soft tissues. However, no change of MG and LG motoneuron excitability was potentially related to no change in the form of muscle fascia and/or somatic components. Our finding was supported by a recent study showing no change in length of MG muscle fascia when diamond deloading tape was applied with and without stretch [30]. Additionally, de Jesus et al. [31] showed no difference of KT effect on quadricep isometric strength and single hop performance in healthy adults when paper-off, 50%, 75%, and 100% KT maximal stretch tensions were applied. These results indicated that the mechanical output of muscle performance is not influenced by KT stretch tensions. In addition, the absent KT effect on sensorimotor inputs via Ia afferent loop observed in the present study may be due to larger individual variation of H-reflex responses in the gastrocnemius, as demonstrated by the large standard deviation in Figure 3. 

Limitations in the present study included that the fact that the H-reflex assessment was conducted during rest in a lying prone position rather than that during any motor activities. The participants recruited in the present study were physically active adults. The absence of corticospinal inputs during the H-reflex assessments may limit practical applications of the study to the control of movement. The resting condition may potentially undermine the acute adaptation of somatosensory inputs by the KT. Secondly, the technique of KT application used in our study was a Y-shaped inhibitory technique. This specific taping technique may not be able to alter the sensorimotor inputs of the triceps surae. Alternative techniques such as the attachment to muscle belly may lead to variants of sensory neural inputs to affect motor performance of the triceps surae. Future studies need to examine whether the location of KT attachment and alternative techniques can provide beneficial effect on motor performance.

## 5. Conclusions

The stretch tension of the KT has minimal influence on motoneuron excitability in the LG and SOL muscles and no influence on the MG muscle during resting conditions. With the KT being widely used in clinical applications and injured individuals going through rehabilitation, it is important that future directions of this research should be pointed to specific populations. Physical therapists usually use KT to facilitate or inhibit an injured muscle; therefore, KT tension may play important roles manipulating motoneuron excitability.

## Figures and Tables

**Table 1:** Physical characteristics of the participants.

Physical Characteristics	Mean ± Standard Deviation (*n* = 12)
Age (years)	21.3 ± 1.2
Height (cm)	175.6 ± 7.1
Body Weight (kg)	69.9 ± 7.1
BMI	22.7 ± 2.3
Soleus muscle length (cm)	37.8 ± 2.0
Lateral gastrocnemius muscle length (cm)	41.5 ± 2.2
Dominant leg (R/L)	11/1
IPAQ (METs)	8658.3 ± 4676.7

BMI, body mass index; IPAQ, international physical activity questionnaire.

**Table 2:** Inter-day reliability of the H_max_, M_max_, and H_max_/M_max_ in pre-KT conditions.

Muscles	H_max_ (mV)		M_max_ (mV)		H_max_/M_max_ (%)	
	ICC (95% CI)	SEM	ICC (95% CI)	SEM	ICC (95% CI)	SEM
Soleus	0.93 (0.81–0.98) nearly perfect	0.055	0.88 (0.68-0.96) very large	0.079	0.91 (0.76–0.97) nearly perfect	0.023
Medial gastrocnemius	0.92 (0.79-0.98) nearly perfect	0.003	0.92 (0.79-0.98) nearly perfect	0.048	0.83 (0.54–0.95) very large	0.002
Lateral gastrocnemius	0.96 (0.90–0.99) nearly perfect	0.022	0.92 (0.81–0.98) nearly perfect	0.118	0.81 (0.48–0.94) very large	0.013

ICC, intraclass correlation coefficients; CI, confident interval; SEM, standard error of measurement; H_max_, peak-to-peak maximal amplitude of H-reflex response; M_max_, peak-to-peak maximal amplitude of M-wave response; H_max_/M_max_, maximal H-reflex and maximal M-wave ratio.

**Table 3:** Modulation of soleus, medial, and lateral gastrocnemius H-reflex during paper-off, 50%, and 100% of maximal kinesiology taping stretch tensions in pre-taping, taping, and post-taping conditions.

Parameters	Paper-Off			50% Maximal Stretch Tension			100% Maximal Stretch Tension		
	Pre-Taping	Taping	Post-Taping	Pre-Taping	Taping	Post-Taping	Pre-Taping	Taping	Post-Taping
Soleus									
H_max_ (mV)	1.82 ± 0.49	1.90 ± 0.49	1.86 ± 0.52	1.89 ± 0.69	1.85 ± 0.72	1.83 ± 0.63	1.70 ± 0.58	1.79 ± 0.64	1.73 ± 0.53
M_max_ (mV)	2.80 ± 0.45	2.79 ± 0.48	2.89 ± 0.46	3.05 ± 0.49 *	2.96 ± 0.44	3.01 ± 0.47	3.02 ± 0.57	2.98 ± 0.54	2.94 ± 0.62
H_max_/M_max_	0.65 ± 0.13	0.68 ± 0.12	0.64 ± 0.13	0.61 ± 0.20	0.62 ± 0.22	0.60 ± 0.19	0.57 ± 0.19	0.60 ± 0.20	0.59 ± 0.17
Medial Gastrocnemius									
H_max_ (mV)	0.48 ± 0.17	0.49 ± 0.16	0.49 ± 0.17	0.46 ± 0.25	0.50 ± 0.24	0.46 ± 0.25	0.45 ± 0.23	0.50 ± 0.25	0.50 ± 0.23
M_max_ (mV)	2.94 ± 1.19	2.86 ± 1.14	2.84 ± 1.26	2.84 ± 1.30	2.72 ± 1.06	2.75 ± 0.94	2.87 ± 1.13	2.65 ± 1.12	2.67 ± 1.14
H_max_/M_max_	0.20 ± 0.12	0.21 ± 0.11	0.21 ± 0.12	0.20 ± 0.11	0.21 ± 0.11	0.19 ± 0.11	0.19 ± 0.13	0.22 ± 0.13	0.22 ± 0.15
Lateral Gastrocnemius									
H_max_ (mV)	0.75 ± 0.39	0.72 ± 0.41	0.69 ± 0.37 **	0.67 ± 0.43	0.66 ± 0.42	0.63 ± 0.38	0.70 ± 0.38	0.72 ± 0.40	0.74 ± 0.37
M_max_ (mV)	3.22 ± 1.54	3.04 ± 1.59	3.09 ± 1.67	2.81 ±1.33	2.64 ± 1.23	2.84 ± 1.32	3.02 ± 1.38	2.84 ± 1.37	2.97 ± 1.20
H_max_/M_max_	0.28 ± 0.19	0.34 ± 0.30	0.34 ± 0.33	0.31 ± 0.19	0.28 ± 0.18	0.31 ± 0.23	0.31 ± 0.23	0.33 ± 0.21	0.30 ± 0.18

* Significant difference between paper-off and 50%KT_max_ (*p* < 0.05). ** Significant difference between pre-taping and post-taping conditions (*p* < 0.05).

## Data Availability

The data are available upon request to the corresponding author’s email.
